# What matters to parents? A scoping review of parents’ service experiences and needs regarding genetic testing for rare diseases

**DOI:** 10.1038/s41431-023-01376-y

**Published:** 2023-06-12

**Authors:** Erin Crellin, Melissa Martyn, Belinda McClaren, Clara Gaff

**Affiliations:** 1 University of Melbourne, Melbourne, VIC Australia; 2 Genomics in Society, Murdoch Children’s Research Institute, Melbourne, VIC Australia; 3 Melbourne Genomics Health Alliance, Walter and Eliza Hall Institute, Melbourne, VIC Australia

**Keywords:** Genetic testing, Paediatrics, Translational research

## Abstract

Patient care experiences are key to promoting better outcomes and are an essential consideration for successful implementation of genomics in paediatric care. To understand parents’ service experiences and needs regarding testing of their child for rare diseases, we conducted a scoping review. Five databases were searched (2000–2022), with 29 studies meeting the inclusion criteria. Experiences of care wholly delivered by genetic services were most commonly reported (*n* = 11). Results were synthesised by mapping extracted data to adapted Picker principles of person-centred care. Parents especially valued and emphasised the importance of feeling ‘cared for’, continuous relationships with clinicians, empathic communication, being kept informed while awaiting genetic test results, linkage with informational and psychosocial resources following results disclosure, and follow-up. Strategies were often proposed by authors to address long-standing unmet needs but evidence from the literature regarding their potential effectiveness was rarely provided. We conclude that ‘what matters’ to parents regarding genetic testing is not dissimilar to other aspects of care. Paediatric medical specialists have existing skill sets, trusted relationships and can apply familiar principles of ‘good’ care to enhance experiences of genetic testing. The lack of evidence for service improvement strategies highlights the pressing need to undertake rigorous design and testing of interventions alongside mainstreaming of genomics into paediatric care.

## Introduction

Exome and genome sequencing (E/GS) promise to provide more timely and accurate diagnoses for children with rare genetic conditions [1]. As the clinical and economic utility of E/GS as an early diagnostic investigation is increasingly recognised by healthcare funders, test availability in clinical settings is increasing [2, 3]. Genetic services are correspondingly struggling to meet demand [4]. To improve patient access, test ordering needs to expand beyond genetic services into mainstream clinical practice [5]. However, effecting practice change in healthcare is notoriously complex and challenging [6]. Interventions are needed to facilitate change and enhance care delivery [7], with understanding the perspectives and needs of diverse stakeholders, including patients and families, critical to identifying and developing effective interventions.

The importance of partnering with patients (and in paediatrics, parents) to help shape and improve service delivery to best meet their needs is increasingly recognised in healthcare [8, 9], evidenced by the inclusion of patient experience in contemporary quality of care standards [10, 11]. A core aspect of this work involves firstly understanding ‘what matters’ to patients (parents) in terms of service delivery [12]. What are their needs, and are they being met? As a first step to addressing these questions and to inform key considerations for service design and intervention development, we reviewed evidence about parents’ service experiences and needs in relation to genetic testing for rare diseases. To the best of our knowledge, the synthesis presented is the first to map experiences of care processes. Recent reviews have focused on singular aspects of care experiences such as understanding of information delivered by providers [13], or interrelated topics such as parent-reported outcomes [14], barriers to genetic test access [15], and the broader supportive care needs of parents caring for children with rare genetic conditions [16]. In mapping the current evidence landscape related to experiences of service provision as a whole, our review identifies opportunities for future intervention research to enhance parents’ care experiences throughout the patient journey and promote health and well-being outcomes [17, 18] in turn.

## Methods

### Methodology

A scoping review of peer-reviewed literature was undertaken. Scoping reviews serve a range of purposes, including identifying key factors related to a concept and gaps in understanding, and narrowing questions to address in subsequent systematic reviews [19]. Their iterative nature is a key methodological strength, in that it enables researchers to refine and clarify the concept of interest while engaging with the literature. A variety of guidance for conducting scoping reviews exist, with our approach guided by the Joanna Briggs Institute’s framework [20] and the Preferred Reporting Items for Systematic Reviews and Meta-Analyses scoping review extension [21].

### Literature search strategy

The literature search was developed under the guidance of an experienced medical librarian. Keyword and MeSH terms from relevant papers were incorporated into the search, with the refined search then run across five databases (Medline, Embase, PubMed, PsycInfo, Web of Science) on October 26, 2021. The search was also rerun on July 29, 2022 to identify any studies published since the initial search was conducted. Search terms included parents and caregivers, genetic and genomic tests, delivery of healthcare, experience, patient satisfaction, patient-centred care, and health service needs. The full search strategy is available in Supplementary Material 1. Records retrieved were imported into Endnote X9 (ref. [22]) for duplicate removal, then Rayyan [23] for title/abstract screening.

### Study selection

Inclusion and exclusion criteria, mapped to the Joanna Briggs Institute’s Population, Concept, Context mnemonic [20], are summarised in Table 1. In brief, the study population is parents of children with rare conditions of suspected monogenic origin. Studies solely focused on experiences of being offered testing for the following were excluded due to being considered of limited utility in considering service needs for genome-wide, germline sequencing in mainstream settings. Paediatric cancers, as treatment-targeted testing and somatic mutations are the main focus; non-syndromic hearing loss, as targeted gene testing is used in the first instance and has a high diagnostic yield; [24] and autism spectrum disorder, a primarily multifactorial condition for which E/GS is not currently considered an appropriate investigation [25]. The experiences of adolescents were excluded due to their distinct nature [26] and our focus on exploring testing for children. The study concept defines experience as experience of care processes (i.e., delivery of care by health professionals), mirroring the way in which experience is defined in patient-reported experience measures [27]. The study context is primarily focussed on outpatient settings; studies examining parent experiences of rapid genomic sequencing delivered in acute care settings were excluded due to the unique challenges in this context (e.g., time pressures involved) that warrant review in their own right [28]. The year range (2000-present) aimed to enable comparisons in service experiences and needs across test modalities.

**Table 1:** Inclusion and exclusion criteria.

Inclusion	Exclusion
Population	
• Parents of children with (suspected or confirmed) rare genetic diseases	• Parents of children with isolated autism spectrum disorder or rare cancers or non-syndromic hearing loss
Concept	
• Parents’ service experiences of diagnostic genetic testing, including: • Interactions with different providers (e.g., paediatricians, clinical geneticists) at different stages of the patient journey • Aspects of service provision valued or considered lacking (i.e., areas of unmet need), as reported by parents directly	• Healthcare provider-reported descriptions • Service experiences of prenatal/rapid/ post-mortem/carrier testing or newborn screening; parents’ views or experiences regarding additional findings • Hypothetical testing or service • Explorations of parent experiences of genetic testing that solely describe the experience and/or impact of the diagnostic odyssey, parent expectations, outcomes related to test results (e.g., affective, behavioural responses) • Experience/impact of caring for a child with a rare disease, and parents’ broader supportive care needs
Context	
• Any outpatient healthcare setting • Peer-reviewed empirical studies • 2000-present	• Acute care settings only • Not published in English • No full-text available

To efficiently and rigorously screen the large number of records retrieved, two reviewers (EC, BM) independently reviewed the same subset (10%) of titles and abstracts. Results were compared, with disagreements resolved through discussion and refinements to the selection criteria accordingly made. One reviewer (EC) then applied the refined criteria to the remaining titles and abstracts. Three reviewers independently reviewed all of the full-text records retrieved (EC, BM, MM), with disagreements resolved through discussion and the criteria further refined through this process. Following full-text review, references of included studies were mined, and forward citation searching was conducted in Google scholar.

### Data charting and synthesis

Data charted included study characteristics (country of origin, study aims, methodology, sample, genetic investigation(s) undertaken, service delivery model), parents’ experiences of care delivery, and strategies or interventions proposed (by parents or study authors) to address parents’ service needs. The charting form (an Excel spreadsheet) was piloted on five studies by EC, modified following team discussion (BM, MM), then applied across studies.

As no patient experience measures specific to genetics could be identified, findings were mapped to a generic framework (the Picker Principles of person-centred care) to help synthesise the results. This empirically-derived framework, developed by the Picker Institute, consists of eight core components of care known to be important to patients [29]. The Picker Principles and a variant based upon the same research, the Institute of Medicine framework, have been found to be broadly applicable to a wide range of disease contexts and care settings [10, 12].

The Picker principles were adapted to context and refined through ongoing discussion among members of the research team (EC, MM, BM). One reviewer (EC) then deductively coded parents’ service experiences to the adapted principles (see Table 2), with subcategories inductively generated in tandem to describe the nuances of parents’ experiences and needs. A second reviewer (MM) reviewed the mapping, and refinements were made until consensus was reached. Results are reported narratively.

**Table 2:** Picker principles adapted to context.

Adapted principles	Description
Clear information, communication, and support for managing child’s ongoing care	Any aspects related to the provision of information or communication with health providers throughout the patient journey that influenced parents’ experiences and feelings of being supported to manage their child’s ongoing care.
Partnering with and providing support for families	Extent to which providers involved parents throughout the patient journey and linked parents with services and counselling and peer supports (in contrast to emotional support provided by health providers directly during clinical consultations).
Environmental context	Any aspect of the location or modality in which care was provided, or the personnel or resources available (including time), that influenced parents’ experiences of care.
Timeliness	Parent readiness to investigate a possible genetic cause for their child’s condition (whether genetic testing was offered at the “right time”), and challenges and needs related to waiting for test results.
Smooth transitions and continuity of care	Smoothness of transitions between different health providers/services involved throughout the patient journey, and the influence of continuous relationships and multidisciplinary care on parents’ experiences.
Effective care by trusted professionals	Parents’ confidence and trust in health providers involved, including parent perceptions of providers’ genetic skill-sets.
Emotional support, empathy, and respect	Relational aspects of care that influenced parents’ experiences, including emotional support, empathy and respect afforded to both the child and family by health providers.

## Results

### Study selection and characteristics

**Fig. 1:**
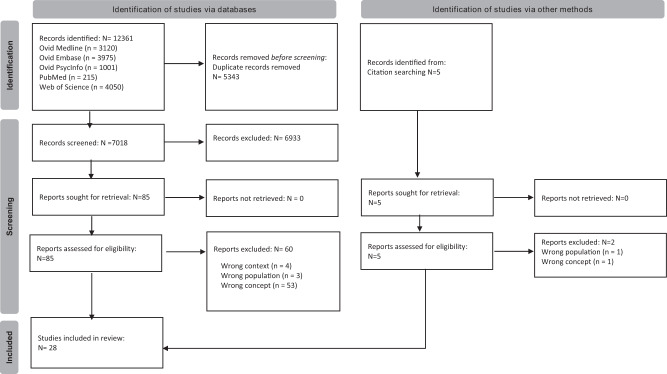
An overview of the screening process for the initial systematic search conducted. As shown in the PRISMA flowchart, 28 of the 7018 records screened post-duplicate removal met the inclusion criteria.

The initial search yielded 12,361 records (see Fig. 1). 7018 titles/abstracts were screened following duplicate removal, with 85 records retrieved for full-text review. Of these, 60 were excluded for reasons detailed in Supplementary Material 2. Three additional studies were included following citation searching. One further study, published since the initial search was run, was identified in the rerun search (Supplementary Fig. S1), generating a total sample of 29 studies for review inclusion.

The majority of studies (17/29) were conducted in North America; [30–46] others originated from Australia [47–52], Ireland [53–55], the Netherlands [56], UK [57], and Dutch Caribbean [58]. Study aims were diverse (see Supplementary Table S1), with most studies (19/29) deploying a qualitative study design [30, 32–39, 42–44, 46, 48, 50, 55–58]. Neurological phenotypes including intellectual disability and developmental delay were common clinical features among children referred for genetic investigation [31, 32, 35, 36, 38, 40–43, 46, 50, 52–55]. Close to half of the studies (14/29) specified the genetic investigation(s) considered or undertaken [35–39, 41–45, 50, 55, 56, 58]. In 10 of these, genome-wide sequencing tests (gene panels, exome or genome sequencing) were conducted, in both research [37, 43–45, 56] and clinical [35, 36, 39, 41, 58] settings.

A range of service delivery models were reported, albeit with varying precision. In 11 studies [33, 34, 36, 37, 41, 42, 48, 52–54, 57], parents were referred to specialised genetics services for genetic assessment and/or testing. In three additional studies [38, 50, 58], some parents initially received their child’s genetic test results from non-genetic paediatric medical specialists (referred to as paediatric medical specialists herein and meaning physicians who have not undertaken specialised training in medical genetics, in contrast to clinical geneticists) prior to genetics referral. Following assessment and pre-test counselling from genetic providers, parents in two studies received genetic test results from paediatric medical specialists and genetic counsellors [43, 45]. In another two studies [39, 49], pre- and post-test counselling was said to have been provided by various health providers including paediatric medical specialists. Paediatric neurologists were involved throughout the patient journey in one study [56], with clinical geneticists playing a supporting role. Paediatric medical specialists were also reported to be involved in another four studies;[31, 40, 51, 55] however the exact nature of their role was unclear. The service delivery model was unspecified or unclear in a further six studies [30, 32, 35, 44, 46, 47].

### Parents’ service experiences

Satisfaction with service delivery and the experience of genetic testing as a whole was reported in eight studies [31, 40–42, 51, 52, 56, 58], two of which used the genetic counselling satisfaction scale [41, 52]. Specific aspects of service delivery parents valued or considered lacking were broadly similar across studies irrespective of differences in service delivery models and the type of genetic investigations conducted. Information-related challenges and needs were most commonly reported followed by (insufficient) support for families during and after results disclosure. A synthesis of the components of care influencing parent service experiences is presented in Table 3 and described below. While mapped separately, a number of these components were interrelated.

**Table 3:** Parents’ service experiences and needs mapped to adapted Picker principles.

Adapted Picker Principles	Sub-domains		Exemplars
Clear information, communication, & support for managing child’s ongoing care	Knowledge & understanding	Perceptions and understanding of information communicated throughout the patient journey [31, 37–39, 44, 47–52, 56, 57]	“I’m just an ordinary mom. (The doctor) levelled with me. He explained to me things step by step, the simplest way, so I would be able to understand things. So that’s very important. This is not our daily language” [44] “We were lost a little bit…We just smiled and went through it…It felt like we were listening to someone speak in another language” [39]
	Affective	Cognitive overload experienced throughout the patient journey, particularly at results disclosure [32, 34–36, 42, 46–50, 53, 57, 58]	“What are your questions, what are your questions? And we didn’t have any … on the day I was feeling a little bit, not shell-shocked or numb, that’s too extreme…. I was processing it… what would have been handy would have been if we’d had a follow-up appointment in maybe a month… It would have given us time to …digest … then go back with informed questions.” [50]
		Impact of the way in which information was communicated on parents’ affective responses [40, 42, 47, 50, 52, 55, 57]	“When any child is diagnosed with any kind of disorder or syndrome health care providers need to understand that at that moment you may be smashing all the dreams the parents have for that [child]… having the person who gives that diagnosis to the parents understand that they are changing that family’s world with the words that are coming out of their mouth may soften the blow” [40]
		Experiences and impact of receiving inconsistent or incorrect information [38, 51]	“[We were] just totally devastated by the initial results… [the neurologist] told me that I had the same duplication as [my child], and he had never seen that before…when I went to the genetics department, they told me it’s actually very common… for six months … I didn’t think there was any hope” [38]
	Sense-making	Challenges in understanding what test results mean for one’s child and family; desire to know ‘next steps’ [30–32, 36, 38, 39, 42, 45, 49, 50, 52, 53, 55, 56, 58]	“I came out of that feeling like, “so what does this mean?” Now what do we do? What is the implication not, dealing with not just, [child’s] own physical health but what does it mean for us” [42]
		Information-seeking behaviours; experiences navigating Internet resources [31, 32, 34, 38–40, 44, 46, 55, 58]	“A lot more people would be at ease if they would get information strictly from an actual doctor or a professional, and links to the pages to read, rather than you trying to Google it.” [44]
		Need for resources to help parents understand & remain abreast of research developments [49, 52]	*Being informed about scientific research was considered important and.… links to understandable and consolidated information [were] desired to help parents to keep up to date*… “like a website… [a] hospital web site. Like where the research comes from, information that we can go read on it on rare genetic kidney disease.” [49]
Partnering with & providing support for families	Provision of psychosocial support	Wanting to be connected with psychosocial supports but such support often not forthcoming [31, 35, 38, 40, 47, 48, 52, 55, 57]	“There was nothing there, no backup, no support, no counselling, just sorry there’s nothing we can do, no offer of help. I did come out of there extremely disappointed…there was no network, even to speak to someone, there was nothing.” [55]
		Need for health providers to ‘check in’ on how parents are faring post-testing [37, 45, 48, 52, 56]	“When families do get the diagnosis of these conditions, they need some help. You got to counsel [them], you have to call them and say right you found out the other day that your daughter/son has got ***, how do you feel? You know and how can we support you?” [37]
		Need for a case manager or equivalent to help guide parents during and after the testing process [34, 40, 48, 53]	*.Several parents also commented on the…need for a* ‘link’ *person to support parents and other family members in the few weeks after the appointment. This would involve a key role in linking them to any new services required, whilst being cognizant of the wider psychological and social impact of receiving genetic information*[53]
	Involvement of parents	Extent to which parents were (or wanted to be) involved [34, 36, 42, 53]	“…when [the geneticist] finally got to the end of what [s/he] was explaining to me and let me talk” [42]
Environmental context	Setting; time & resources available	Influence of aspects of the location or modality in which a diagnosis is delivered, and the supports or personnel present at the time, on parents’ experiences [31, 35, 38, 40, 42, 47, 48, 51, 53, 56]	“Confirmation given over the phone with no support available” [51] “I knew the deletion from when they gave me the results (by phone)…. But I didn’t know what they meant… I was going crazy trying to figure that out… [At the appointment] the charts really helped because I’m more visual” [38]
		Value of and need for adequate time to talk and ask questions [31, 37, 39, 40, 53, 54]	“He let us ask questions, and he let us ask as many questions as we wanted. He didn’t end the conversation. We did” [31]
		Accessibility of physical environment [48]	*Parents reported that small waiting rooms without facilities for entertaining children, or those that did not adequately accommodate mobility devices were another barrier to positive experiences of attending genetic health services* [48]
Timeliness	Parental readiness; waiting for results	Impact of the timing in which testing is offered on parents’ experiences [31, 40, 42, 46]	*Some parents spoke of* “the right time” *for, or being “ready” to receive, the diagnosis. One mother who reported a positive experience recalled: “*maybe, just, we were ready for it” [42]
		Impact of waiting for results; support needs during this time [36, 49, 52, 56, 57]	“Being kept informed is important I think… as waiting for the results, knowing that these can alter your life completely, is hard” [56]
Smooth transitions & continuity of care	Moving between and interacting with different providers	Understanding of the roles and responsibilities of the different providers involved; perceived distinctiveness of genetics [34, 48, 53, 54, 57]	“When the doctor told me, ‘I’m going to send you to a geneticist.’ I said, ‘What is that?’ because I had never even heard of the word ‘geneticist’ before…. I was scared.” [34] *A difference identified by three parents between this appointment and those previously attended was that, at a genetic counselling appointment, it was the parents and the child who were the patients, and not only the child* [53]
		Need for integrated care [32, 49, 52, 57]	*Parents found the lack of liaison between genetics and other departments unhelpful* [57]
	Continuity of care	Value of continuous relationships with providers [35, 38, 42, 44, 49, 52]	*.[A] subset of parents felt more comfortable receiving information from their local paediatrician. Parents who opted for their local paediatrician to be the primary point of care described long-standing rapport and trusted that their paediatrician understood the comprehensive needs of their child*[49]
Effective care by trusted health providers	Confidence & trust	Whether interactions with providers inspire trust and confidence in providers and the information imparted [30, 31, 44, 47, 49–51, 57]	“Very satisfied because everyone involved knew what they were saying and doing” [51] “The geneticist said in her mind that all that we had seen with him fitted in with other kids with this deletion… paediatrician said the same thing so we were happy that… this was the explanation” [50]
Emotional support, empathy & respect	Relational aspects of care	Experiences of receiving empathic & respectful care [31, 33, 34, 39, 42, 44, 47, 49, 51, 54, 56]	“Paediatric ophthalmologist not very sensitive; told us loudly with their back to our daughter… who is old enough to understand.” [47] “I feel like they’ve been really human with me… they’re not just focusing on the illness. They’re focusing on our family.… on our personalities.” [39]
		Responsiveness to parents’ emotional needs [42, 51]	“Doctors don’t know how to handle the shock that sets in with diagnosis” [51]
	CALD considerations	Additional considerations to meet the needs of, and provide culturally safe and respective care to, CALD families [33, 36, 39, 48, 57]	“The father [of my children] finished primary school [only], if [he] asks for information in Spanish they’re going to give it to [him], [and he] will read it, and [he] will not understand anything even if it is in Spanish… I sometimes understand more in English than in Spanish” [33] “Having that Indigenous [support] person next to you makes you feel more comfortable and confident to ask questions and talk… instead of just, yep, which is what a lot of Indigenous people do. They’re just like, yep. Even though they don’t understand…, they go, yep.” [48]

#### Clear information, communication, and support for managing child’s ongoing care

##### Parents want straightforward, jargon-free information

While some parents felt well-informed throughout the patient journey and praised providers’ use of accessible language [31, 37, 44, 47, 49, 51, 52, 56, 57], others commented upon and expressed frustration with the incomprehensible nature of the genetic information providers imparted [31, 39, 42, 48, 54, 56]. Some parents [39, 48] described hiding their lack of understanding behind head nods and smiles, with several reluctant to speak up and ask questions because they found genetics intimidating and felt or feared sounding ‘stupid’ [48, 54]. A number found (or felt they would have found) visuals helpful in aiding their understanding [38, 44, 49], as well as lay summaries that could additionally be shared with family members, educators and health providers [44, 49, 52].

##### Parents find it difficult to absorb information during test appointments

Parents frequently described feeling overwhelmed at different stages of the genetic testing process, most notably at time of diagnosis [32, 34–36, 42, 46–50, 53, 57, 58]. As a result, many found it difficult to absorb and process information during appointments [32, 34–36, 46, 48–50, 57, 58]. Parents found (or indicated they would have found) take-away resources helpful along with the opportunity for follow-up to ask questions that only become apparent with the benefit of processing time [44, 45, 48, 50, 57]. A need to reduce the volume of information communicated to parents and focus on what parents are most interested in – implications for their child’s care – was also emphasised in several studies of parents whose children underwent genome-wide sequencing [39, 44, 45].

##### Parents struggle to use and make sense of genetic results

Across studies, challenges in making sense of what genetic test results mean for one’s child and family were commonly described [30, 32, 38, 39, 42, 45, 49, 50, 52, 53, 55, 56, 58]. Sense-making was an ongoing process for a number of parents, with inherently practical questions regarding implications for their child’s immediate care and future outlook continuing to be raised weeks to months after initial results disclosure [45, 53, 55]. Parents wanted to know how to use the genetic information they received and what the ‘next steps’ were [30, 31, 36, 42, 45, 49, 56], and many expressed frustration and disappointment with the inability of health professionals to provide the clarity and guidance they desired [32, 42, 50, 55, 56].

To fill this void of information, parents frequently turned to the Internet despite often being advised by health professionals not to do so [31, 32, 34, 38–40, 44, 46, 55, 58]. Internet-searching experiences were mixed in general [30, 31, 38, 39, 43, 46, 51, 55], whether it be for contextualising the meaning of genetic test results [34, 38, 39, 58], searching for possible diagnoses [30], or deciphering the information health professionals imparted [31, 58]. Some felt worse off after reading worst-case scenarios, with Internet searches not always based on accurate information [38, 46, 55]. Some parents expressed a need for health professionals to provide curated resources or links to reputable websites instead of parents being left to their own devices [40, 44]. Parents in two studies [49, 52] expressed a related need for tools to help them understand and keep up-to-date with research developments.

##### Impact of communication on parents’ affective responses

The potential for communication with providers to both exacerbate and ameliorate the affective responses parents experienced throughout the patient journey was commonly highlighted [40, 42, 47, 50, 52, 55, 57]. The way in which information was framed was particularly influential, with some parents reporting feeling hurt or shattered by providers’ blunt communication style and (poor) choice of words [40, 42, 55]. Parents emphasised the importance of providing hope, even in the face of considerable challenges and uncertainties [40, 42, 55]. Parents’ ability to cope and adapt to uncertainty was also enhanced when health professionals acknowledged the novelty and evolving nature of genomic information [50].

The emotional consequences of receiving incorrect information from paediatric medical specialists during initial results disclosure was highlighted in two studies [38, 51], with a mother in one [38] describing how she spent six weeks needlessly stressed about her child’s variant of uncertain significance while awaiting a consequent genetics appointment.

#### Involvement and support for families/carers

##### Parents want to be linked with formal and informal psychosocial supports post genetic testing

Parents wanted health providers to connect them with genetic support groups, other families, and/or counselling supports following results disclosure [31, 35, 38, 40, 47, 48, 52, 55, 57], and were often frustrated by health providers’ failure to facilitate these connections [35, 38, 55, 57]. Indeed, some parents felt let down, lost, and isolated as a result [35, 55, 57]. The value of offering or linking families with psychosocial support at the time of genetic diagnosis was highlighted in two studies [40, 51], with parents more likely to report positive or satisfactory experiences as a result. Some parents considered follow-up an important emotional support in and of itself following results disclosure [37, 45, 48, 52, 56]. They felt that having someone check in on parents during this emotionally challenging and often confusing period of time would be invaluable for helping parents cope and connect with supports as needed. In addition to these needs, parents in several studies [34, 40, 48, 53] expressed a need for a case manager or ‘link person’ to help parents connect with and navigate health services post-testing.

##### Partnering with parents is important but not all want to be involved to the same degree

Parents in a few studies expressed frustration with having a passive role when interacting with health providers throughout their child’s patient journey [34, 42, 53]. Specifically, some felt dismissed, removed from clinical decision-making, and that conversations were driven by health providers’ agendas rather than their own [34, 42, 53]. Not all parents perceived a passive role negatively, however. Some parents in a US study exploring Latinx parents’ experiences of genome-wide sequencing were comfortable with the genetic test decision-making process being clinician-driven given their self-assessed lack of understanding [36].

#### Environmental context

Various environmental factors influenced parents’ service experiences [31, 35, 38, 40, 42, 47, 48, 51, 53, 56]. Experiences of receiving genetic test results by telephone rather than in-person were often less positive [38, 40, 51], largely due to misunderstandings arising or the limited psychosocial support available in such instances. Personnel or resources present were also influential [38, 40, 47, 53, 56], with the involvement of genetic counsellors associated with more positive diagnostic experiences in one study [40], for example. In another study [48], the extent to which consultation rooms accommodated families with disabilities and other additional needs influenced parents’ experiences of care. Having sufficient time available to talk and ask questions was also important to parents [31, 37, 39, 40, 53, 54], with several disappointed when genetic consultations did not afford them this opportunity [31, 40, 53].

#### Timeliness

##### Timing in which testing is offered impacts parents’ experiences

Parental readiness to receive and process a genetic diagnosis influenced parents’ service experiences in several studies [31, 40, 42, 46]. Readiness was a function of time for some parents, with parents in one study [46] reflecting that they felt better prepared to face genetic testing after having time to adapt to their child’s intellectual disability and additional needs. Accordingly, more positive experiences were reported by parents whose children were older at time of genetic diagnosis in two studies [31, 40].

The importance of parents being able to decline the offer of genetic testing was also highlighted in one study [46], with parents describing the immense relief they experienced when, after being encouraged by physicians to consider genetic testing for their child, another health provider empowered them to decline the test offer.

##### Long wait times impact parents’ experiences

Long waits inherent to many genetic investigations negatively impacted parents service experiences in several studies [36, 49, 52, 56, 57]. Indeed, the wait for test results was described as one of the most challenging aspects of the genetic testing process in two studies [36, 57]. Parents emphasised the importance of being supported and kept informed by health providers during the stressful waiting period [52, 56, 57], the value of which was highlighted [52, 57].

#### Smooth transitions and continuity of care

##### Transitions between different providers involved in child’s testing journey are disjointed for many

Parents referred to genetic services were often confused, concerned and even fearful about what the appointment would entail [34, 48, 53, 54, 57]. Some felt unprepared due to receiving minimal information on what to expect from their referring provider [48, 53, 54]. Parents built-up the appointment in their minds as a result of their lack of understanding and a perception that genetics was ‘different’ from other health services [53, 54]. This perceived distinctiveness was affirmed following the appointment, with some parents citing the nature of the examination conducted and fact that both the child and the parents were ‘patients’ as examples [53, 54]. In addition to disjointed transitions *to* genetics, disjointed transitions post genetic testing were described in several studies [42, 50, 53], with parents often unclear as to who was responsible for follow-up. A related need for integrated, multidisciplinary care was emphasised [32, 49, 52, 57], with both positive [32, 49] and negative [52, 57] experiences of these noted.

##### Value of continuous relationships with health providers

The value of continuous relationships with health providers in enhancing parents’ service experiences, decision-making, understanding, and feelings of being supported was highlighted in several studies [35, 38, 42, 44, 49, 52]. Of note in two [38, 49], familiar providers such as paediatricians were considered helpful or best placed to help parents contextualise and make sense of their child’s genetic test results.

#### Effective care by trusted professionals

##### Confidence and trust in health providers influences parents’ experiences and behaviours

The importance of interactions with health providers inspiring confidence and trust was highlighted [30, 31, 44, 47, 49–51, 57], with parents more likely to report positive experiences when they felt like they were ‘in the hands of the experts’ [43, 47, 49]. Relatedly, more positive experiences were reported when non-genetic providers were open about their limited knowledge of certain genetic conditions and referred parents on [31]. The consequences of parents lacking confidence in providers’ genetics skill sets were described in a few studies [30, 54], with some parents expressing reluctance to ask questions or resorting to their own means to find out information.

Interrelated to confidence in providers, confidence in the information parents received was reported in two studies [50, 52], with confidence fostered when congruent explanations were provided by paediatric medical specialists and genetic providers and hampered when parents perceived providers to be withholding information.

#### Emotional support, empathy, and respect

Relational aspects of care were important to parents [31, 33, 34, 39, 42, 44, 47, 49, 51, 54, 56], with positive [31, 39, 42, 44, 49, 51, 52, 54, 56] and negative [42, 47, 51] experiences of receiving respectful and empathic care throughout their child’s patient journey described. In particular, parents placed great value on feeling ‘cared for’ by providers [33, 39, 44, 49, 56], and the ability of providers to respond to their emotional needs during genetics consultations [42, 51].

#### Additional needs of culturally and linguistically diverse (CALD) families

Specific needs and experiences of parents from CALD communities were reported in a subset of studies [33, 36, 39, 48, 58]. Two studies [33, 36] described additional challenges comprehending genetic information, often due to reliance upon medical translators and varying literacy levels. Of note, some parents in these studies stressed the importance of not assuming parents prefer to receive and best understand information communicated in their native language [33, 36]. Several studies [33, 48, 58] also highlighted the importance of receiving culturally safe and respectful care. Caregivers in one study reporting the genetic service experiences of Aboriginal and Torres Strait Islander peoples [48] described their discomfort interacting with providers of the opposite gender, and the consequent impact on their ability to comprehend information. Some felt the support of an indigenous health provider throughout the patient journey would have made them feel more at ease and aided their understanding.

### Finding a better way: proposals to meet parent needs

Authors proposed strategies to improve service delivery in 19/29 studies [31–34, 38, 40, 43–46, 49–53, 55–58]. These strategies were targeted at the level of health services [31, 34, 49, 53, 56], providers [31, 33, 38, 40, 44, 50–52, 56–58], and parents [33, 34, 38, 40, 44–46, 49, 51–53, 58]. Most proposed strategies were intuitive suggestions from study authors [32, 33, 38, 43, 45, 46, 49–52, 55–58], with only one citing evidence for the effectiveness of certain strategies suggested (e.g., two-tiered disclosure appointments) [31]. Examples of intuitive strategies included education and training for health professionals [33, 38, 40, 50, 51, 55, 56], enhanced emotional and practical support during and following appointments [32, 34, 38, 40, 43–45, 49–52, 56, 57], and access to informational resources [34, 44, 46, 49, 52, 58].

## Discussion

As a first step to considering how genomics care in paediatric settings can be designed to enhance patient (parent) experiences, we reviewed evidence about parents’ service experiences and needs in relation to genetic testing for rare genetic diseases. Key aspects of care considered important to parents were identified, with these aspects broadly mapping to an established framework, the Picker principles of person-centred care [12, 29]. The fact that principles well-known to be important to patients such as empathic communication and support, feeling listened to and heard, were highly valued by parents suggests that in the eyes of parents, ‘good’ care in genetics is not dissimilar from ‘good’ care in healthcare in general [59]. Others have also (albeit indirectly) drawn this parallel when commenting upon the utility of pre-eminent breaking bad news frameworks to communicating paediatric genetic test results [31, 40, 60]. These parallels should be reassuring to paediatric medical specialists. While the content of the service delivered may arguably be more complex, drawing upon their existing skill sets and applying familiar care principles will likely go a long way to enhancing parents’ care experiences throughout the patient journey.

Nonetheless, several challenges and needs specific to genetic testing were identified. The wait for genetic test results (genomic sequencing results especially) is often considerably longer than other diagnostic investigations. Studies report that parents desire to be supported and kept informed during this anxious time, although exactly what this would look like in practice was often unclear. Sense-making was also highly challenging for many parents due to the inherently complex and often-uncertain nature of their child’s test result. Much has been written about the need to appraise and acknowledge these uncertainties with families to facilitate adaptation [61, 62]. Paediatric medical specialists may be well-placed to do so, with prognostic uncertainty an inherent aspect of many developmental disability diagnoses, for example. Insights from the growing use of genomics in acute settings [28] may also help shed light on how clinicians working in outpatient clinics can be supported to help parents make sense of their child’s test result.

Given the consistency in which sense making challenges (and related frustrations) were reported across studies, the integration of E/GS into paediatrics also presents an opportunity to unpack this long-standing unmet need further. Parents’ desire for practical guidance and more information (a need which often cannot be met presently) may speak more to an underlying need for greater emotional support than information per se [62]. The UK Medical Research Council’s widely regarded guidance on developing complex interventions [63] highlights the importance of comprehensively understanding the problem an intervention seeks to address, from multiple perspectives. Adoption of this guidance in future intervention research will increase the likelihood of more effective and sustainable interventions being developed.

While the fact that aspects of care important to parents were broadly similar regardless of the type of genetic test delivered or model of care provided suggests our review findings are informative for mainstream service design, it is worth noting the relative scarcity of studies describing genetics care provided by paediatric medical specialists. Experiences of genomic sequencing delivered in clinical (i.e., non-research) settings were similarly scarce. The time and resources available in these settings differ, highlighting the need for further research and pragmatic research designs to better understand how service delivery can be best supported. It is encouraging that the service experiences and perspectives of parents from CALD backgrounds have begun to be examined. Future research should expand upon the small subset of studies included in this review to ensure genomic sequencing in paediatric settings is delivered in a way that meets the needs of diverse populations.

Given this review has affirmed the universal nature of core care components important to patients (parents) and exposed the lack of evidence to support proposed strategies, future research could develop a generic tool to help match identified needs to (evidenced-based) intervention categories. Possible benefits of such a tool include improving the ability to identify existing successful interventions that could be repurposed [64], and gaps where new interventions are needed.

To help design better solutions which fit local contexts, codesign with broad stakeholder input should be an integral feature of future intervention development. Encouragingly, some researchers have already begun to use codesign to build upon their exploratory work included in this review [65], with the acceptability of the parent supports developed enhanced as a result [66]. Future systematic reviews should examine how interventions are designed and the relationship between the (co)design process and intervention outcomes to enable successful approaches to be replicated elsewhere.

### Limitations

The findings of this review need to be considered alongside its limitations. The search was limited to English-language publications. Some relevant literature may also have been missed despite our efforts to include a broad range of MeSH and keyword terms, with terms used to denote care experiences notoriously heterogeneous [17]. The Picker principles of person-centred care were an imperfect fit for a paediatric context having been developed from empirical research with adult patients in acute care settings [67]. Other frameworks such as the measure of processes of care [68] were reviewed, however challenges remain when applied to a genetics context where both the child and their parents are often ‘patients’. It is worth noting the many similarities of these frameworks and therefore using an alternate framework would likely have had limited impact on the synthesis of results. Nonetheless, the comprehensiveness of the Picker framework was a key strength, with aspects such as timeliness not captured in the measure of processes of care domains, for example.

## Conclusion

Our review provides insight into how care can be designed to enhance patient (parent) experiences as the availability of E/GS in paediatric settings increases. Mainstreaming of genomics into medical care presents an opportunity to address long-standing unmet needs and improve how interventions are identified and developed. While further research in more diverse clinical settings is needed, paediatric medical specialists should find it reassuring that current evidence suggests ‘what matters’ to parents regarding genetic testing is not dissimilar to other services. Drawing upon their existing skill sets and applying familiar principles of ‘good’ care will likely go a long way to enhancing parents’ care experiences with genomics and their health and well-being outcomes in turn.

## Supplementary information

Supplementary material

## References

[1] Wright CF, Fitzpatrick DR, Firth HV (2018). Paediatric genomics: diagnosing rare disease in children. Nat Rev Genet.

[2] Sachdev R, Field M, Baynam GS, Beilby J, Berarducci M, Berman Y (2021). Paediatric genomic testing: navigating Medicare rebatable genomic testing. J Paediatr Child Health.

[3] Lewis C, Buchannan J, Clarke A, Clement E, Friedrich B, Hastings-Ward J, et al. Mixed-methods evaluation of the NHS Genomic Medicine Service for paediatric rare diseases: study protocol. NIHR Open Res. 2021; 10.3310/nihropenres.13236.1. 10.3310/nihropenres.13236.1 PMC7612282 35098132

[4] Fennell AP, Hunter MF, Corboy GP (2020). The changing face of clinical genetics service delivery in the era of genomics: a framework for monitoring service delivery and data from a comprehensive metropolitan general genetics service. Genet Med.

[5] Burton H, Hall A, Kroese M, Raza S. Genomics in mainstream clinical pathways. PHG Foundation; 2017. https://www.phgfoundation.org/report/genomics-mainstream-clinical-pathways. Accessed 29 Sept 2022.

[6] Bauer MS, Damschroder L, Hagedorn H, Smith J, Kilbourne AM. An introduction to implementation science for the non-specialist. BMC Psychol. 2015; 10.1186/s40359-015-0089-9. 10.1186/s40359-015-0089-9 PMC4573926 26376626

[7] Skivington K, Matthews L, Simpson SA, Craig P, Baird J, Blazeby JM (2021). A new framework for developing and evaluating complex interventions: update of Medical Research Council guidance. BMJ.

[8] Bombard Y, Baker GR, Orlando E, Fancott C, Bhatia P, Casalino S, et al. Engaging patients to improve quality of care: a systematic review. Implement Sci. 2018; 10.1186/s13012-018-0784-z. 10.1186/s13012-018-0784-z PMC6060529 30045735

[9] Bate P, Robert G (2006). Experience-based design: from redesigning the system around the patient to co-designing services with the patient. Qual Saf Health Care.

[10] Institute of Medicine. Crossing the quality chasm: a new health system for the 21st century. Natl Acad Press, Washington DC, 2001. 25057539

[11] Australian Commission on Safety and Quality in Health Care. Patient-centred care: improving quality and safety through partnerships with patients and consumers, ACSQHC, Sydney, 2011.

[12] Robert G, Cornwell J What matters to patients? Developing the evidence base for measuring and improving patient experience. The King’s Fund, London, 2011.

[13] Gereis J, Hetherington K, Ha L, Robertson EG, Ziegler DS, Barlow-Stewart K (2022). Parents’ understanding of genome and exome sequencing for pediatric health conditions: a systematic review. Eur J Hum Genet.

[14] Hayeems RZ, Luca S, Assamad D, Bhatt A, Ungar WJ (2021). Utility of genetic testing from the perspective of parents/caregivers: a scoping review. Children.

[15] Best S, Vidic N, An K, Collins F, White SM (2022). A systematic review of geographical inequities for accessing clinical genomic and genetic services for non-cancer related rare disease. Eur J Hum Genet.

[16] Pelentsov LJ, Laws TA, Esterman AJ (2015). The supportive care needs of parents caring for a child with a rare disease: a scoping review. Disabil Health J.

[17] Doyle C, Lennox L, Bell D (2013). A systematic review of evidence on the links between patient experience and clinical safety and effectiveness. BMJ Open.

[18] McAllister M, Payne K, MacLeod R, Nicholls S, Donnai D, Davies L (2008). What process attributes of clinical genetics services could maximise patient benefits?. Eur J Hum Genet.

[19] Munn Z, Peters MDJ, Stern C, Tufanaru C, McArthur A, Aromataris E. Systematic review or scoping review? Guidance for authors when choosing between a systematic or scoping review approach. BMC Med Res Methodol. 2018; 10.1186/s12874-018-0611-x. 10.1186/s12874-018-0611-x PMC6245623 30453902

[20] The Joanna Briggs Institute. Methodology for JBI scoping reviews. The Joanna Briggs Institute, South Australia, Australia, 2015.

[21] Tricco AC, Lillie E, Zarin W, O’Brien KK, Colquhoun H, Levac D (2018). PRISMA extension for scoping reviews (PRISMA-ScR): checklist and explanation. Ann Intern Med.

[22] Endnote. Version X9. Thomson Reuters, USA, 2018.

[23] Ouzzani M, Hammady H, Fedorowicz Z, Elmagarmid A. Rayyan—a web and mobile app for systematic reviews. Syst Rev. 2016; 10.1186/s13643-016-0384-4. 10.1186/s13643-016-0384-4 PMC5139140 27919275

[24] Sung V, Downie L, Paxton GA, Liddle K, Birman CS, Chan WW (2019). Childhood hearing Australasian medical professionals network: consensus guidelines on investigation and clinical management of childhood hearing loss. J Paediatr Child Health.

[25] Amor DJ (2018). Investigating the child with intellectual disability. J Paediatr Child Health.

[26] Wainstein T, Marshall SK, Ross CJ, Virani AK, Austin JC, Elliott AM, et al. Experiences with genetic counseling, testing, and diagnosis among adolescents with a genetic condition: a scoping review. JAMA Pediatr. 2022; 10.1001/jamapediatrics.2021.4290. 10.1001/jamapediatrics.2021.4290 34807246

[27] Goodrich J, Fitzsimons B (2019). Capturing patient experience to improve healthcare services. Nurs Stand.

[28] Lynch F, Nisselle A, Stark Z, Gaff CL, McClaren B (2021). Parents’ experiences of decision making for rapid genomic sequencing in intensive care. Eur J Hum Genet.

[29] The Picker Institute Europe. The Picker principles of person-centred care. 2022. https://picker.org/who-we-are/the-picker-principles-of-person-centred-care/.

[30] Barton KS, Wingerson A, Barzilay JR, Tabor HK (2019). “Before Facebook and before social media…we did not know anybody else that had this”: parent perspectives on internet and social media use during the pediatric clinical genetic testing process. J Community Genet.

[31] Demarest S, Marsh R, Treat L, Fisher MP, Dempsey A, Junaid M (2022). The lived experience of parents’ receiving the diagnosis of cdkl5 deficiency disorder for their child. J Child Neurol.

[32] Glassford MR, Purcell RH, Pass S, Murphy MM, Bassell GJ, Mulle JG (2022). Caregiver perspectives on a child’s diagnosis of 3q29 deletion: “we can’t just wish this thing away. J Dev Behav Pediatr.

[33] Hallford H, Coffman MA, Obregon-Tito AJ, Morales AH, Williamson Dean L (2020). barriers to genetic services for Spanish-speaking families in states with rapidly growing migrant populations. J Genet Couns.

[34] Hernandez VR, Selber K, Tijerina MS (2006). Visioning family-centered care in genetics: what parents and providers have to say. J Genet Couns.

[35] Li X, Nusbaum R, Smith-Hicks C, Jamal L, Dixon S, Mahida S (2019). Caregivers’ perception of and experience with variants of uncertain significance from whole exome sequencing for children with undiagnosed conditions. J Genet Couns.

[36] Luksic D, Sukhu R, Koval C, Cho MT, Espinal A, Rufino K (2020). A qualitative study of Latinx parents’ experiences of clinical exome sequencing. J Genet Couns.

[37] McConkie-Rosell A, Pena LDM, Schoch K, Spillmann R, Sullivan J, Hooper SR (2016). Not the end of the odyssey: parental perceptions of whole exome sequencing (WES) in pediatric undiagnosed disorders. J Genet Couns.

[38] Reiff M, Bernhardt BA, Mulchandani S, Soucier D, Cornell D, Pyeritz RE (2012). “What does it mean?”: uncertainties in understanding results of chromosomal microarray testing. Genet Med.

[39] Watnick D, Odgis JA, Suckiel SA, Gallagher KM, Teitelman N, Donohue KE (2021). “Is that something that should concern me?”: a qualitative exploration of parent understanding of their child’s genomic test results. Hum Genet Genom Adv.

[40] Waxler JL, Cherniske EM, Dieter K, Herd P, Pober BR (2013). Hearing from parents: The impact of receiving the diagnosis of Williams syndrome in their child. Am J Med Genet A.

[41] Wynn J, Ottman R, Duong J, Wilson AL, Ahimaz P, Martinez J (2018). Diagnostic exome sequencing in children: A survey of parental understanding, experience and psychological impact. Clin Genet.

[42] Ashtiani S, Makela N, Carrion P, Austin J (2014). Parents’ experiences of receiving their child’s genetic diagnosis: a qualitative study to inform clinical genetics practice. Am J Med Genet A.

[43] Inglese CN, Elliott AM (2019). CAUSES Study, Lehman A. New developmental syndromes: Understanding the family experience. J Genet Couns.

[44] Li KC, Birch PH, Garrett BM, MacPhee M, Adam S, Friedman JM (2016). Parents’ perspectives on supporting their decision making in genome-wide sequencing. J Nurs Scholarsh.

[45] Liang NSY, Adam S, Elliott AM, Siemens A, du Souich C, CAUSES Study. (2022). After genomic testing results: parents’ long-term views. J Genet Couns.

[46] Makela NL, Birch PH, Friedman JM, Marra CA (2009). Parental perceived value of a diagnosis for intellectual disability (ID): a qualitative comparison of families with and without a diagnosis for their child’s ID. Am J Med Genet A.

[47] Anderson M, Elliott EJ, Zurynski YA. Australian families living with rare disease: experiences of diagnosis, health services use and needs for psychosocial support. Orphanet J Rare Dis. 2013; 10.1186/1750-1172-8-22. 10.1186/1750-1172-8-22 PMC3599672 23398775

[48] Dalach P, Savarirayan R, Baynam G, McGaughran J, Kowal E, Massey L, et al. “This is my boy’s health! Talk straight to me!” perspectives on accessible and culturally safe care among Aboriginal and Torres Strait Islander patients of clinical genetics services. Int J Equity Health. 2021; 10.1186/s12939-021-01443-0. 10.1186/s12939-021-01443-0 PMC8052687 33865398

[49] Nevin S, McLoone J, Wakefield CE, Kennedy S, McCarthy H (2022). Genetic testing in the pediatric nephrology clinic: understanding families’ experiences. J Pediatr Genet.

[50] Wilkins EJ, Archibald AD, Sahhar MA, White SM (2016). “It wasn’t a disaster or anything”: parents’ experiences of their child’s uncertain chromosomal microarray result. Am J Med Genet A.

[51] Zurynski Y, Deverell M, Dalkeith T, Johnson S, Christodoulou J, Leonard H, et al. Australian children living with rare diseases: experiences of diagnosis and perceived consequences of diagnostic delays. Orphanet J Rare Dis. 2017; 10.1186/s13023-017-0622-4. 10.1186/s13023-017-0622-4 PMC5387276 28399928

[52] Nevin SM, Wakefield CE, Barlow-Stewart K, McGill BC, Bye A, Palmer EE (2022). Psychosocial impact of genetic testing on parents of children with developmental and epileptic encephalopathy. Dev Med Child Neurol.

[53] Barr O, Millar R (2003). Parents of children with intellectual disabilities: their expectations and experience of genetic counselling. J Appl Res Intellect Disabil.

[54] Barr O, McConkey R (2007). A different type of appointment: the experiences of parents who have children with intellectual disabilities referred for genetic investigation. J Res Nurs.

[55] Fitzgerald J, Wilson C, Kelly C, Gallagher L (2021). ‘More than a box of puzzles’: understanding the parental experience of having a child with a rare genetic condition”. Eur J Med Genet.

[56] Krabbenborg L, Schieving J, Kleefstra T, Vissers LELM, Willemsen MA, Veltman JA (2016). Evaluating a counselling strategy for diagnostic WES in paediatric neurology: an exploration of parents’ information and communication needs. Clin Genet.

[57] Skirton H (2006). Parental experience of a pediatric genetic referral. MCN Am J Matern Nurs.

[58] Verberne EA, van den Heuvel LM, Ponson-Wever M, de Vroomen M, Manshande ME, Faries S (2022). Genetic diagnosis for rare diseases in the Dutch Caribbean: a qualitative study on the experiences and associated needs of parents. Eur J Hum Genet.

[59] Levetown M and the Committee on Bioethics. Communicating with children and families: from everyday interactions to skill in conveying distressing information. Pediatrics . 2008;121:e1441–60.. 10.1542/peds.2008-0565 18450887

[60] Witt MM, Jankowska KA (2018). Breaking bad news in genetic counseling—problems and communication tools. J Appl Genet.

[61] Parascandola M, Hawkins JS, Danis M (2002). Project MUSE - patient autonomy and the challenge of clinical uncertainty. Patient Auton Chall Clin Uncertain.

[62] Lipinski SE, Lipinski MJ, Biesecker LG, Biesecker BB (2006). Uncertainty and perceived personal control among parents of children with rare chromosome conditions: the role of genetic counseling. Am J Med Genet C Semin Med Genet.

[63] O’Cathain A, Croot L, Duncan E, Rousseau N, Sworn K, Turner KM (2019). Guidance on how to develop complex interventions to improve health and healthcare. BMJ Open.

[64] Waltz TJ, Powell BJ, Fernández ME, Abadie B, Damschroder LJ. Choosing implementation strategies to address contextual barriers: diversity in recommendations and future directions. Implement Sci. 2019; 10.1186/s13012-019-0892-4. 10.1186/s13012-019-0892-4 PMC6489173 31036028

[65] Nevin SM, Wakefield CE, Dadich A, LeMarne F, Macintosh R, Beavis E (2022). Hearing parents’ voices: a priority-setting workshop to inform a suite of psychological resources for parents of children with rare genetic epilepsies. PEC Innov.

[66] Nevin SM, Wakefield CE, Le Marne F, Beavis E, Macintosh R, Sachdev R (2022). Piloting positive psychology resources for caregivers of a child with a genetic developmental and epileptic encephalopathy. Eur J Paediatr Neurol.

[67] Gerteis M, Edgman-Levitan S, Daley J, Delbanco TL. Through the patient’s eyes: understanding and promoting patient-centered care. Jossey-Bass, San Francisco, 1993.

[68] Cunningham BJ, Rosenbaum PL (2014). Measure of processes of care: a review of 20 years of research. Dev Med Child Neurol.

