# Supplementary material for: What matters to parents? A scoping review of parents’ service experiences and needs regarding genetic testing for rare diseases
Source: Eur J Hum Genet. 2023 Jun 12;31(8):869–78. doi: 10.1038/s41431-023-01376-y (PMC10400618; doi:10.1038/s41431-023-01376-y)
Supplement: Supplementary file 1 — Supplementary material [file 41431_2023_1376_MOESM1_ESM.docx]

# Supplementary Material 1

Search conducted in five electronic databases (Medline, Embase, PsycInfo**,** PubMed and Web of Science) on 26/10/2021 and 29/07/2022.

## Medline

1. exp *parents/ or *caregivers/

2. (mother? or father? or parent* or caregiver* or care-giver*).tw,kf.

3. 1 or 2

4. *Genetic Testing/

5. *Exome/ or *genome/

6. exp *Whole Genome Sequencing/

7. *High-Throughput Nucleotide Sequencing/

8. *Oligonucleotide Array Sequence Analysis/

9. Chromosomal-microarray.tw,kf.

10. Variant-of-Uncertain-Significance.tw,kf.

11. *Precision Medicine/

12. *Genetic Services/

13. exp *Genetics, Medical/

14. *sequence analysis, DNA/ or exp *Genetic Predisposition to Disease/

15. ((clinical-exome or genome-wide) adj sequencing).tw,kf.

16. (precision-medicine or personali#ed-medicine or WES or exom* or genom* or genetic-test* or genetic-counsel?ing or genetic-diagnos*).tw,kf.

17. 4 or 5 or 6 or 7 or 8 or 9 or 10 or 11 or 12 or 13 or 14 or 15 or 16

18. *Physician-Patient Relations/ or *professional-family relations/ or *professional-patient relations/

19. *"Delivery of Health Care"/

20. (Experience* or Perception* or view? or opinion* or support* or need? or communicat* or satisfaction or dissatisfaction).tw,kf.

21. exp *Patient Satisfaction/

22. *Patient-Centered Care/ or *patient navigation/

23. *"Health Services Needs and Demand"/ or *Needs Assessment/

24. *Communication/ or *communication barrier/

25. *Health Knowledge, Attitudes, Practice/

26. exp *"Surveys and Questionnaires"/ or exp *qualitative research/

27. *Decision Making/

28. 18 or 19 or 20 or 21 or 22 or 23 or 24 or 25 or 26 or 27

29. exp Prenatal diagnosis/ or Neonatal screening/ or exp Neoplasms/ or genetic carrier screening/

30. 3 and 17 and 28

31. 30 not 29

32. limit 31 to (english language and yr="2000 -Current")

33. limit 32 to (case reports or comment or editorial or guideline or letter or practice guideline)

34. 32 not 33

35. (exp animals/ or exp plants/ or (plant or plants or rat or rats or mouse or mice or rodent* or swine or porcine or murine or sheep or lamb or lambs or pig or pigs or piglet or piglets or rabbit or rabbits or cat or cats or dog or dogs or cattle or bovine or monkey or monkeys or trout or marmoset or marmosets or fly or flies or yeast or drosophila or nematode or worm or worms or roundworm* or frog or frogs or xenopus or zebrafish or zebra-fish).ti.) not human*.sh.

36. 34 not 35

## Embase

1. exp *parent/ or *caregiver/

2. (mother? or father? or parent* or caregiver* or care-giver*).tw,kf,dq.

3. 1 or 2

4. genetic screening/

5. Exome/ or genome/

6. exp whole genome sequencing/ or whole exome sequencing/

7. high throughput sequencing/

8. DNA microarray/ or single nucleotide polymorphism array/

9. Chromosomal-microarray.tw,kf,dq.

10. Variant-of-Uncertain-Significance.tw,kf,dq.

11. Personalized medicine/

12. genetic service/

13. Genetic Counseling/

14. medical genetics/

15. DNA sequencing/ or genetic predisposition/

16. ((clinical-exome or genome-wide) adj sequencing).tw,kf,dq.

17. (precision-medicine or personali#ed-medicine or WES or exom* or genom* or genetic-test* or genetic-counsel?ing or genetic-diagnos*).tw,kf,dq.

18. 4 or 5 or 6 or 7 or 8 or 9 or 10 or 11 or 12 or 13 or 14 or 15 or 16 or 17

19. doctor-patient relationship/ or human-relation/ or professional-patient relationship/

20. health care delivery/

21. (Experience* or Perception* or view? or opinion* or support* or need? or communicat* or satisfaction or dissatisfaction).tw,kf,dq.

22. Patient satisfaction/

23. patient care/

24. health care need/ or needs assessment/

25. interpersonal communication/ or communication barrier/

26. attitude to health/

27. health care survey/ or questionnaire/ or qualitative research/

28. decision-making/

29. 19 or 20 or 21 or 22 or 23 or 24 or 25 or 26 or 27 or 28

30. 3 and 18 and 29

31. exp prenatal diagnosis/ or newborn screening/ or exp neoplasms/ or (genetic-carrier-screening or carrier-screening).tw,kf,dq.

32. 30 not 31

33. limit 32 to (english language and yr="2000 -Current")

34. limit 33 to (note or letter or books or chapter or conference abstract or conference paper or conference review or editorial)

35. case report/

36. 33 not (34 or 35)

37. (plant or plants or rat or rats or mouse or mice or rodent* or swine or porcine or murine or sheep or lamb or lambs or pig or pigs or piglet or piglets or rabbit or rabbits or cat or cats or dog or dogs or cattle or bovine or monkey or monkeys or trout or marmoset or marmosets or fly or flies or yeast or drosophila or nematode or worm or worms or roundworm* or frog or frogs or xenopus or zebrafish or zebra-fish).ti. and animal experiment/

38. (Animal experiment/ or exp plant/) not (human experiment/ or human/)

39. 36 not (37 or 38)

## PsycInfo

1. exp *parents/ or *caregivers/

2. (mother? or father? or parent* or caregiver* or care-giver*).ti,ab,id.

3. 1 or 2

4. *Genetic Testing/

5. *Genome/

6. *Genomic sequencing/

7. (high-throughput-nucleotide-sequencing or next-generation-sequencing).ti,ab,id.

8. Chromosomal-microarray.ti,ab,id.

9. *Precision medicine/

10. *Genetic Counseling/

11. *Genetics/ or *genomics/ or medical-genetic*.ti,ab,id.

12. Genetic-predisposition-to-disease.ti,ab,id.

13. (genetic* adj service*).ti,ab,id.

14. Variant-of-uncertain-significance.ti,ab,id.

15. ((clinical-exome or genome-wide) adj sequencing).ti,ab,id.

16. (precision-medicine or personali#ed-medicine or WES or exom* or genom* genomic-test* or genetic-test* or genetic-counsel?ing or genetic-diagnos*).ti,ab,id.

17. 4 or 5 or 6 or 7 or 8 or 9 or 10 or 11 or 12 or 13 or 14 or 15 or 16

18. *Client satisfaction/

19. (doctor-patient-relation* or doctor-family-relation* or physician-patient-relation* or physician-family-relation* or professional-patient-relation* or professional-family-relation*). ti,ab,id.

20. *Interpersonal communication/ or *communication barriers/ or *interpersonal interaction/ or *communication/

21. *Health care delivery/

22. *Patient-Centered Care/

23. *Health service needs/ or *needs assessment/

24. *Health knowledge/ or *health attitudes/

25. Decision Making/

26. exp *Qualitative methods/ or *questionnaires/

27. (Experience* or Perception* or view? or opinion* or support* or need? or communicat* or satisfaction or dissatisfaction).ti,ab,id.

28. 18 or 19 or 20 or 21 or 22 or 23 or 24 or 25 or 26 or 27

29. 3 and 17 and 28

30. Prenatal diagnosis/ or exp neoplasms/ or (newborn-screening or neonatal-screening or genetic-carrier-screening or carrier-screening).ti,ab,id.

31. 29 not 30

32. limit 31 to (english language and yr="2000-current")

33. limit 32 to peer-reviewed journal

34. (animal not human).po.

35. 33 not 34

## PubMed – Articles not indexed in MEDLINE, e-pubs, articles ahead of print

| **Contents** | **Search string** |
| --- | --- |
| Parents/caregivers  #1 | **Title/Abstract**  “Parent*” OR “caregiver*” OR “care-giver*” OR “mother” OR “mothers” OR “father” OR “fathers” |
| Genetic/genomic testing  #2 | **Title/Abstract**  “Genetic-test*” OR “precision-medicine” OR “personalized-medicine” OR “personalised-medicine” OR “DNA-sequence-analysis” OR “high-throughput-nucleotide-sequencing” OR “next-generation-sequencing” OR “WES” OR “chromosomal-microarray” OR “exome*” OR “genom*” OR “genetic-counselling” OR “genetic-counseling” OR “genetic-diagnos*” OR “genetic-service*” OR “genetics-service*” OR “medical-genetic*” OR “variant-of-uncertain-significance” OR “genetic-predisposition-to-disease” OR “DNA-sequencing” |
| Health service experiences and needs  #3 | **Title/Abstract**  “Patient-centred-care” OR “patient-centered-care” OR “satisfaction” OR “dissatisfaction” OR “experience*” OR “perception*” OR “view” OR “views” OR “opinion*” OR “support*” OR “need” OR “needs” OR “communicat*” OR “delivery-of-healthcare” OR “delivery-of-health-care” OR “health-care-delivery” OR “healthcare-delivery” OR “health-care-need*” OR “healthcare-need*” OR “health-service-need*” OR “health-services-need*” OR “needs-assessment*” OR “doctor-patient-relation*” OR “doctor-family-relation*” OR “physician-patient-relation*” OR “physician-family-relation*” OR “professional-patient-relation*” OR “professional-family-relation*” OR “health-attitude*” OR “health-knowledge*” OR “decision-making” OR “survey” OR “surveys” OR “questionnaire*” OR “qualitative” |
| #4 | **All fields**  NOTNLM OR publisher[sb] OR inprocess[sb] OR pubmednotmedline[sb] OR indatareview[sb] OR pubstatusaheadofprint |
| #5 | #1 AND #2 AND #3 AND #4 |
| Pre-natal  #6 | **Title/Abstract**  “Pre-natal-diagnos*” OR “prenatal-diagnos*” OR “prenatal-test*” OR “pre-natal-test*” OR “newborn-screening” OR “neonatal-screening” OR “carrier-screening” OR “cancer*” OR “neoplasm*” OR “tumor*” OR “tumour*” |
| #7 | #5 NOT #6 |
| #8 | #7 limited to English, 2000-present, NOT books and documents, case reports, comment, editorial, guideline, letter, practice guideline |
|  | **All fields**  ("Animal” OR "animals” OR “plant” OR “plants” OR "rat” OR "rats” OR "mouse” OR "mice” OR "rodent*” OR "swine” OR "porcine” OR "murine” OR "sheep” OR "lamb” OR "lambs” OR "pig” OR "pigs” OR "piglet” OR "piglets” OR "rabbit” OR "rabbits” OR "cat” OR "cats” OR "dog” OR "dogs” OR "cattle” OR "bovine” OR "monkey” OR "monkeys” OR "trout” OR "marmoset” OR "marmosets” OR "fly” OR "flies” OR "yeast” OR "drosophila” OR "nematode” OR "worm” OR "worms” OR "roundworm*” OR "frog” OR "frogs” OR "xenopus” OR "zebrafish” OR "zebra-fish") NOT ("human” OR "humans” OR "patient” OR "patients” OR "newborn*” OR "baby” OR "babies” OR "neonat*” OR "infan*” OR "toddler*” OR "pre schooler*” OR "preschooler*” OR "kindergarten” OR "boy” OR "boys” OR "girl” OR "girls” OR "child” OR "children” OR "childhood” OR "adolescen*” OR "pediatric*” OR "paediatric*” OR "youth*” OR "teen” OR "teens” OR "teenage*” OR "school aged*” OR "school child*” OR "school girl*” OR "school boy*” OR "schoolgirl*” OR "schoolboy*” OR "man” OR "men” OR "woman” OR "women” OR "adult” OR "adults” OR "middle age*” OR "elderly") |
| #9 | #8 NOT #9 |

## Web of Science

| **Contents** | **Search string – TITLE search** |
| --- | --- |
| #1 | **Title**  (Parent* OR caregiver* OR care-giver* OR mother OR mothers OR father OR fathers) AND (Genetic-test* OR genomic-test* OR precision-medicine OR personalized-medicine OR personalised-medicine OR genomic-sequencing OR DNA-sequence-analysis OR high-throughput-nucleotide-sequencing OR next-generation-sequencing OR WES OR chromosomal-microarray OR exome* OR genom* OR genetic-counselling OR genetic-counseling OR genetic-diagnos* OR medical-genetic* OR genetic-service* OR genetics-service* OR variant-of-uncertain-significance OR genetic-predisposition-to-disease OR DNA-sequencing OR single-nucleotide-polymorphism-array) AND (Patient-centred-care OR patient-centered-care OR satisfaction OR dissatisfaction OR experience* OR perception* OR view OR views OR opinion* OR support* OR need OR needs OR communicat* OR delivery-of-healthcare OR delivery-of-health-care OR health-care-delivery OR healthcare-delivery OR health-care-need* OR healthcare-need* OR health-service-need* OR health-services-need* OR needs-assessment* OR doctor-patient-relation* OR doctor-family-relation* OR physician-patient-relation* OR physician-family-relation* OR professional-patient-relation* OR professional-family-relation* OR health-attitude* OR health-knowledge* OR decision-making OR survey OR surveys OR questionnaire* OR qualitative) |
| #2 | **Title**  Pre-natal-diagnos* OR prenatal-diagnos* OR prenatal-test* pre-natal-test* OR newborn-screening OR neonatal-screening OR carrier-screening OR cancer* OR neoplasm* OR tumor* OR tumour* OR ((Animal OR animals OR plant OR plants OR rat OR rats OR mouse OR mice OR rodent* OR swine OR porcine OR murine OR sheep OR lamb OR lambs OR pig OR pigs OR piglet OR piglets OR rabbit OR rabbits OR cat OR cats OR dog OR dogs OR cattle OR bovine OR monkey OR monkeys OR trout OR marmoset OR marmosets OR fly OR flies OR yeast OR drosophila OR nematode OR worm OR worms OR roundworm* OR frog OR frogs OR xenopus OR zebrafish OR zebra-fish) NOT (human OR humans OR patient OR patients OR newborn* OR baby OR babies OR neonat* OR infan* OR toddler* OR pre schooler* OR preschooler* OR kindergarten OR boy OR boys OR girl OR girls OR child OR children OR childhood OR adolescen* OR pediatric* OR paediatric* OR youth* OR teen OR teens OR teenage* OR school aged* OR school child* OR school girl* OR school boy* OR schoolgirl* OR schoolboy* OR man OR men OR woman OR women OR adult OR adults OR middle age* OR elderly)) |
| #3 | #1 NOT #2 |
| Limits applied | Limit #3 to English, 2000-present, NOT case report, letters, editorial materials, meeting |

| **Contents** | **Search string – ABSTRACT search** |
| --- | --- |
| #1 | **Abstract**  (Parent* OR caregiver* OR care-giver* OR mother OR mothers OR father OR fathers) AND (Genetic-test* OR genomic-test* OR precision-medicine OR personalized-medicine OR personalised-medicine OR genomic-sequencing OR DNA-sequence-analysis OR high-throughput-nucleotide-sequencing OR next-generation-sequencing OR WES OR chromosomal-microarray OR exome* OR genom* OR genetic-counselling OR genetic-counseling OR genetic-diagnos* OR medical-genetic* OR genetic-service* OR genetics-service* OR variant-of-uncertain-significance OR genetic-predisposition-to-disease OR DNA-sequencing OR single-nucleotide-polymorphism-array) AND (Patient-centred-care OR patient-centered-care OR satisfaction OR dissatisfaction OR experience* OR perception* OR view OR views OR opinion* OR support* OR need OR needs OR communicat* OR delivery-of-healthcare OR delivery-of-health-care OR health-care-delivery OR healthcare-delivery OR health-care-need* OR healthcare-need* OR health-service-need* OR health-services-need* OR needs-assessment* OR doctor-patient-relation* OR doctor-family-relation* OR physician-patient-relation* OR physician-family-relation* OR professional-patient-relation* OR professional-family-relation* OR health-attitude* OR health-knowledge* OR decision-making OR survey OR surveys OR questionnaire* OR qualitative) |
| #2 | **Abstract**  Pre-natal-diagnos* OR prenatal-diagnos* OR prenatal-test* pre-natal-test* OR newborn-screening OR neonatal-screening OR carrier-screening OR cancer* OR neoplasm* OR tumor* OR tumour* OR ((Animal OR animals OR plant OR plants OR rat OR rats OR mouse OR mice OR rodent* OR swine OR porcine OR murine OR sheep OR lamb OR lambs OR pig OR pigs OR piglet OR piglets OR rabbit OR rabbits OR cat OR cats OR dog OR dogs OR cattle OR bovine OR monkey OR monkeys OR trout OR marmoset OR marmosets OR fly OR flies OR yeast OR drosophila OR nematode OR worm OR worms OR roundworm* OR frog OR frogs OR xenopus OR zebrafish OR zebra-fish) NOT (human OR humans OR patient OR patients OR newborn* OR baby OR babies OR neonat* OR infan* OR toddler* OR pre schooler* OR preschooler* OR kindergarten OR boy OR boys OR girl OR girls OR child OR children OR childhood OR adolescen* OR pediatric* OR paediatric* OR youth* OR teen OR teens OR teenage* OR school aged* OR school child* OR school girl* OR school boy* OR schoolgirl* OR schoolboy* OR man OR men OR woman OR women OR adult OR adults OR middle age* OR elderly)) |
| #3 | #1 NOT #2 |
| Limits applied | Limit #3 to English, 2000-present, NOT case report, meeting, letters, editorial materials, patent, news |

# Supplementary Material 2

Exclusion criteria hierarchy used for full-text screening and detailed reasons for exclusion.

**E1** – Full-text irretrievable (2021-10-26: N=0; 2022-07-29: N=0)

**E2** – Wrong context (2021-10-26: N=4; 2022-07-29: N=0)

**E2a** Non-English (n=1)

**E2b** Outside year range (n=1)

**E2c** Conference proceeding, review, commentary (n=2)

**E2d** Acute care setting only (n=0)

**E3** – Wrong population (2021-10-26: N=3; 2022-07-29: N=1)

**E3a** Parents of children with cancer (n=0, n=0)

**E3b** Parents of children with multi-factorial conditions (n=3, n=0)

**E3c** Parents of children with non-syndromic sensorineural hearing loss (n=0, n=1)

**E4** – Wrong concept (2021-10-26: N=53; 2022-07-29: N=4)

**E4a** Health professionals’ views only (n=0, n=0)

**E4b** Other types of testing or service (n=12, n=1)

**E4c** Hypothetical diagnostic genetic/genomic testing or service (n=7, n=0)

**E4d** Expectations; personal utility, outcomes related to test results (e.g., affective or behavioural responses, family communication of genetic info); experience and/or impact of diagnostic odyssey (n=18, n=2)

**E4e** Experience/impact of caring for a child with a rare disease and/or broader supportive care needs (n=7, n=1)

**E4f** Evaluation of genetic counselling and related interventions delivered by genetic counsellors (n=9, n=0)

# Figure S1. PRISMA diagram for updated search.

**Previous studies**

**Identification of new studies via databases**

Studies included in previous version of review: N = 28

Records identified: N=15048

Ovid Medline (n=3675)

Ovid Embase (n=4296)

Ovid PsycInfo (n=1065)

PubMed (n=1697)

Web of Science (n=4315)

Records removed *before screening*:

Duplicate records removed:

N = 13997

**Identification**

Total studies included in review:

N = 29

Reports assessed for eligibility:

N = 7

Reports sought for retrieval:

N = 7

Records screened: N = 1051

Records excluded: N = 1044

Reports not retrieved: N = 0

**Screening**

Reports excluded: N=6

Wrong population (n=1)

Wrong concept (n=4)

Study previously identified via citation searching (n=1)

New studies included in review:

N = 1

**Included**

# Table S1. Characteristics of included studies.

| **Author, year** | **Country** | **Study aim** | **Study design** | **Study sample** | **Test modality** | **Model of care** |
| --- | --- | --- | --- | --- | --- | --- |
| Anderson et al., 2013 [47] | Australia | To examine the health, psychosocial, and financial impacts of rare diseases on families | Quantitative (survey incl. open-ended responses) | 30 parents (24 mothers, 6 fathers) of children with lysosomal storage or mitochondrial genetic disorders; all children had a specific genetic diagnosis | Unspecified | Unclear (referral to a metabolic genetics service but unknown whether genetic diagnosis was delivered by other providers prior to referral) |
| Ashtiani et al., 2014 [42] | Canada | To explore the genetics service experiences of parents | Qualitative | 13 parents (10 mothers, 3 fathers) of 10 index children with DD^1^; all children received a genetic diagnosis | Karyotype  /FISH or CMA | 3 families were referred to genetics service for results disclosure. All other families were referred to genetics for testing. 2 families received diagnosis while child was an inpatient |
| Barr and Miller, 2003 [53] | Ireland | To explore parents’ expectations of and experiences interacting with genetics services | Mixed methods | 11 parents (7 mothers, 4 fathers) of children with DD; decision to pursue or outcome of genetic testing unspecified | Unspecified | Referral to a clinical genetics service |
| Barr and McConkey, 2007 [54] | Ireland | To investigate parents’ experiences of being referred to a genetics service | Mixed methods | 19 parents (12 mothers, 7 fathers) of children with ID^2^ | Unspecified (‘genetic investigations continuing’ for children of 10 parents) | Referral to a clinical genetics service by a paediatrician or neurologist |
| Barton et al., 2019 [30] | USA | To explore parents’ experiences of Internet and social media usage throughout the genetic testing process | Qualitative | 21 families (18 mothers, 2 fathers) of children who had undergone clinical genetic testing; all but 4 children had a genetic diagnosis | Unspecified | Unspecified |
| Dalach et al., 2021 [48] | Australia | To explore the genetics service experiences of Aboriginal and Torres Strait Islander people | Qualitative | 63 patients or carers attending genetics services in 3 Australian states; most (49/63) were attending for investigation of a rare disease. All 4 men interviewed were parents /guardians of a child with a suspected rare disease. | Unspecified | Referral to a clinical genetics service |
| Demarest et al., 2022 [31] | USA | To investigate parents' experiences receiving a diagnosis of CDKL5 deficiency disorder (CDD) for their child | Mixed methods | 37 parents of children with CDD interviewed; results compared with responses of 345 families included in the International CDD database who completed diagnostic experience section of a questionnaire. | Unspecified | Unspecified, however results indicate various providers were involved (‘parents reported a range of positive and negative experiences across all provider disciplines (geneticist, neurologist, genetic counsellor, or general paediatrician)’) |
| Fitzgerald et al., 2021 [55] | Ireland | To explore how parents of children with rare genetic conditions interpret, adapt to, and cope with their child's diagnosis | Qualitative | 30 caregivers (24 mothers, 6 fathers) of 26 children, most of whom had a clinical diagnosis of DD/ID. De novo mutations were identified in 18/26, inherited mutation in 6; status of 2 unknown | CMA | Authors note in the Discussion: 'the referral for testing and the subsequent initial communication of the result was often completed by health care professionals outside the field of genetics' |
| Glassford et al., 2022 [32] | USA | To explore caregiver views of the impact of a diagnosis of 3q29Del in their child | Qualitative | 15 mothers of children with 3q29Del syndrome | Unspecified | Unspecified |
| Hallford et al., 2020 [33] | USA | To investigate Spanish-speaking parents' genetics knowledge, awareness of genetics services, and experiences accessing and navigating these services | Qualitative | 29 parents of children with defined (n=27) or suspected (n=1) genetic conditions | Unspecified | Referral to a genetics service in 3 different US states |
| Hernandez et al., 2006 [34] | USA | To explore consumer and service provider  views on the delivery of genetics services in a US state, including their suggestions for how service delivery could be improved to better meet consumer needs | Qualitative | 28 consumers, 38 genetics service providers. Consumers were primarily female (22/28) and were family members of a child, adolescent, or adult with a suspected or diagnosed chromosomal, metabolic, or genetic-based disorder | Unspecified | Referral to a genetics service |
| Inglese et al., 2019 [43] | Canada | To explore families’ experiences when a novel genetic syndrome is diagnosed in their child or sibling | Qualitative | 12 family members (8 mothers, 3 fathers, 1 sibling) of 9 children, most of whom had ID/DD and dysmorphic anomalies. Pathogenic variants were identified in 5/9, likely pathogenic variants in 2, 2 received a VUS 'considered consistent with a probable clinical diagnosis'. | Genome-wide sequencing (research setting) | Clinical evaluation conducted by a clinical geneticist, pre-test counselling by a genetic counsellor. Post-test counselling provided to families by referring physician and research study genetic counsellor |
| Krabbenborg et al., 2016 [56] | Netherlands | To investigate the experiences and needs of parents in relation to the genetic counselling process for exome sequencing | Qualitative | 24 parents (9 couples, 6 single parents) of 15 children with complex, unexplained neurological disorders with a suspected genetic origin. 6/15 received a definitive genetic diagnosis, 5 a lead for a possible genetic diagnosis, no genetic cause identified in 4. | Trio exome sequencing (research setting) | Neurologists provided pre-test counselling; opportunity for parents to then ask additional questions from a clinical geneticist. Neurologists disclosed results. Prior to results disclosure, neurologists and clinical geneticists worked together to compile any information available about the genetic condition, which families could then access via a website. |
| Li et., 2016 [44] | Canada | To investigate the experiences and needs of parents in relation to considering genome-wide sequencing for their child | Qualitative | 15 parents (12 mothers, three fathers) of 15 children with suspected genetic disorders. 3/15 children had received a genetic diagnosis via GWS at time of interview, 10 remained undiagnosed, while 2 parents did not respond when questioned | Genome-wide sequencing (research setting) | Unspecified |
| Li et., 2018 [35] | USA | To investigate the psychosocial impact of receiving a VUS for their child, and identify factors influencing adaption | Qualitative | 15 parents (3 fathers, 12 mothers) of 14 children who had received a VUS. Children had a range of clinical features, many of which were neurological. | Exome sequencing | Unclear. Study indicates exome sequencing completed 'through the Department of neurology and neurogenetics' |
| Liang et al., 2022 [45] | Canada | To investigate levels of decisional regret among parents, and their views and experiences of pursuing genome-wide sequencing for their child several months after results disclosure | Mixed methods | 121 parents of children with suspected genetic disorders undertook the decisional regret scale. 32 parents were interviewed (31 mothers, 1 father). 17/32 children received a genetic diagnosis; 15 remained undiagnosed | Trio exome or genome sequencing (research setting) | Clinical evaluation conducted by a clinical geneticist, pre-test counselling by a genetic counsellor. Post-test counselling provided to families by referring physician and/or a genetic counsellor |
| Luksic et al., 2020 [36] | USA | To investigate Latinx parents' experiences of exome sequencing | Qualitative | 38 Latinx parents (1 father, 37 mothers; 22/38 only spoke Spanish) of children with a range of clinical features including autism, DD, seizures. Pathogenic variants identified in 13/38; VUS in 7, 18 children received a negative result | Exome sequencing | Families were referred to genetics for testing in all but one case; this family was referred to genetics for results disclosure. An interpreter was involved in all or part of the genetics consultation for 21 parents. |
| Makela et al., 2009 [46] | Canada | To investigate the value families place upon receiving a genetic diagnosis for their child's intellectual disability | Qualitative | 20 parents of children with (n = 10) and without (n = 10) a precise genetic diagnosis for their child's ID | Unspecified | Unclear, although referral to a clinical genetics service implied from recruitment description (recruited from 'the provisional medical genetics program') |
| Nevin et al., 2022 [49] | Australia | To explore parents' experiences of the testing process, the psychosocial impact of test results, and parents’ informational needs and preferences | Mixed methods | 26 parents (19 mothers, 7 fathers) of 25 children with renal disease of suspected genetic origin. 13/25 children received a genetic diagnosis; 11/25 remained undiagnosed. 1 child was yet to be tested | Unspecified | Counselling provided to families by treating nephrologist, within a renal genetics clinic, or through referral to a clinical genetics service |
| Nevin et al., 2022 [52] | Australia | To explore parents' experiences of genetic testing, the psychosocial impact of genetic testing, and parents’ informational and support needs | Mixed methods | 25 parents (20 mothers, 5 fathers) of 25 children with developmental and epileptic encephalopathies. 17/25 children received a genetic diagnosis; 8/25 remained diagnosed | Unspecified | Referral to a clinical genetics service |
| McConkie-Rosell et al., 2016 [37] | USA | To explore ‘key factors contributing to the process of empowerment’ | Qualitative | 19 parents (16 mothers, 3 fathers) of 19 children with ‘undiagnosed disorders’. 11/19 children received a definitive/likely diagnosis, 3 a possible diagnosis. 5/19 remained undiagnosed | Trio exome sequencing (research setting) | Pre-test and post-test counselling provided by clinical geneticists and genetic counsellors |
| Reiff et al., 2012 [35] | USA | To explore how families comprehend and make meaning of CMA results, and their informational and support needs | Qualitative | 31 parents (23 mothers, 8 fathers) of 25 children with a range of phenotypes including ID/DD, autism, congenital anomalies. 11/25 children received a pathogenic result, 14 a VUS | CMA | For 15 families, results were disclosed by a clinical geneticist or genetic counsellor; other families were referred to a genetics health professional by ordering non-geneticist following initial results disclosure. |
| Skirton, 2000 [56] | UK | To explore parents’ experiences being referred to a genetics service | Qualitative | 20 parents of children with suspected or confirmed genetic disorders | Unspecified | Referral to a clinical genetics service |
| Verberne et al., 2022 [58] | Dutch Caribbean | To explore parents’ experiences receiving a genetic diagnosis for their child, and their genetics service needs | Qualitative | 30 parents of 24 children (including one twin) with a confirmed genetic diagnosis of a rare disease. 11/24 children had ID | 10/24 underwent gene panels, 7 single gene tests, 3 CMA, 2 methylation analysis, 2 multiple genetic tests | Referring paediatrician usually present during genetics appointment. Diagnosis often initially disclosed by paediatrician due to infrequency of visiting geneticist’s visits |
| Watnick et al., 2022 [39] | USA | To explore parents’ experiences of genomic testing, in particular their understanding of genetic test results | Qualitative | 24 parents (22 mothers, 2 fathers) of 22 children with neurological, cardiac or immunological disorders of suspected genetic origin. 5/22 received a positive result, 10 a negative test result, 7 received a VUS | Exome sequencing, gene panels or CMA | Pre and post-test counselling provided by a genetic counsellor, clinical geneticist or a non-genetic medical professional |
| Waxler et al., 2013 [40] | USA | To investigate parents’ experiences receiving a diagnosis of William syndrome in their child | Quantitative (survey incl. open-ended responses) | 439 caregivers of children diagnosed with Williams syndrome | Unspecified | Unclear but range of health professionals indicated as being involved in child's patient journey, most frequently genetics health professionals and paediatricians |
| Wilkins et al., 2016 [50] | Australia | To explore parents’ experiences receiving an uncertain CMA result for their child | Qualitative | 9 parents (7 mothers, 2 fathers) of 8 children with DD/ID, autism, multiple congenital anomalies. De novo microdeletions were identified in 5/8, inherited microdeletions in 3 | CMA | For 5 families, results were initially returned by non-geneticist clinicians and referral to a clinical geneticist followed. 2 families received results from clinical geneticists directly. 1 family recalled receiving result in mail followed by appointments with ordering non-geneticist and a geneticist |
| Wynn et al., 2017 [41] | USA | To explore parents’ experiences and understanding of exome sequencing | Quantitative | 192 parents (168 mothers, 24 fathers). Most children (64%) had a neurological aspect to their condition. 79/192 children received a positive diagnostic result (4 of which were identified via CMA conducted in parallel), 78 a negative result, while 35 received a VUS | Exome sequencing | Children referred to clinical genetics for testing in both in- and outpatient settings |
| Zurynski et al., 2017 [51] | Australia | To investigate parents’ experiences of seeking and receiving a genetic diagnosis for their child | Quantitative (survey incl. open-ended responses) | 462 caregivers (89% mothers, 8.5% fathers; less than 1% foster carers or grandmothers) of children with rare diseases. 428/462 children had received a definitive genetic diagnosis | Unspecified | Unclear, however parents reported ‘paediatricians, geneticists & neurologists most often [being] the first health professional to raise the possibility of the diagnosis, and to confirm the final diagnosis’ |

^1^DD = Developmental delay;^2^ID = Intellectual disability
